# A Method for the Rapid Measurement of Alkylresorcinols in Flour, Bread and Related Products Based on ^1^H qNMR

**DOI:** 10.3390/foods9081025

**Published:** 2020-07-31

**Authors:** Athina Tsirivakou, Eleni Melliou, Prokopios Magiatis

**Affiliations:** Department of Pharmacy, Laboratory of Pharmacognosy and Natural Products Chemistry, National and Kapodistrian University of Athens, 15771 Athens, Greece; a.tsiriv@gmail.com (A.T.); emelliou@pharm.uoa.gr (E.M.)

**Keywords:** alkylresorcinols, qNMR, wheat, cereals, flour

## Abstract

The main objectives of the current work were to investigate differences among flours from traditionally preserved Greek varieties of cereals, and especially those of wheat, and in parallel, to correlate those potential differences with the presence of bioactive natural ingredients. In this context, we developed a new, fast, and simple method for the measurement of total 5-alkylresorcinols in cereals and related foods by qNMR. Several types of flour (white or whole-grain) coming from wheat, i.e., *Triticum dicoccum, T. monococcum, T. aestivum, T. durum* and *T. turgidum,* corn, barley, rye and oat from a certified producer in Greece were used either as raw materials or for the production of bread, pasta and flakes. A small portion of the flour or the corresponding product was extracted with DMSO-d_6_. The liquid part was directly analyzed by NMR (400 MHz). The simplicity of the NMR spectrum of the total extract and the lack of overlapping peaks permitted the development of a high throughput quantitative method for the measurement of total bioactive alkylresorcinols in less than 15 min. Grains, whole grain flours and breads from old varieties of *T. dicoccum* and *T.monococcum* showed high contents of alkylresorcinols (455–1148 mg/Kg), while the same compounds were completely absent from white flour and the corresponding bread. The term high-phenolic flour is proposed to distinguish among flour types.

## 1. Introduction

Wheat (*Triticum* spp.), olive trees and grapevines are considered to be the three basic plants of the Mediterranean diet; in particular, wheat is the most important plant in the history of human nutrition in Europe and the Middle East. Although famous bioactive small molecules like resveratrol or oleocanthal have been discovered in grape and olive oil, wheat products are not similarly known, from the consumer’s perspective, as sources of health-protective ingredients. However, alkylresorcinols (ARS), an important class of phytochemicals found in wheat, have attracted the interest of scientists in the last thirty years [1,2,3]. 

Alkylresorcinols, 1,3-dihydroxy-5-n-alkylbenzenes, are phenolic lipids found in some plant families, as well as in some algae, sponges, fungi and bacteria [2]. The main nutritional sources of alkylresorcinols are whole grain, bran of wheat and rye, with contents that may reach 1–4 g/kg of dry material [4,5,6,7,8,9,10]. ARS are adequately absorbed [11] and have exhibited interesting bioactivities in vitro, including direct antioxidative properties, lipoxygenase inhibition, inhibition of copper-induced LDL oxidation, DNA-strand scission, colon and prostate cancer cell growth inhibition and inhibition of lipase activity in the adipose tissue cells [3,12,13]. Despite being present in high amounts in specific cereals like wheat and rye, and despite their reported bioactivities and potential role in health protection, their concentration is not widely used for the determination of grain quality or the quality of the final food products. For example, the term high-phenolic olive oil has been used as a marketing description in recent years based on the EU health claim [14] and the big variability in the phenolic content of olive oil; however, nothing similar has happened for cereals. One possible reason for this is the limited data from human nutritional intervention studies, and another is the rather complex analytical methodologies required for the selective measurement of alkylresorcinols in cereal products [4,5,6,7,8,9,10,15,16,17,18,19,20].

To overcome the challenges associated with analytical methodologies, and based on previous successful applications of high-throughput analyses of bioactive small molecules in several foods (olive oil [21], beer [22], wine [23]) or botanic extracts [24,25,26], our target is to develop a fast and simple method for the measurement of total 5-alkylresorcinols in cereals and related foods (bread, pasta, wafers etc.) by qNMR. The developed method requires a total time of less than 15 min per sample, without the use of standards, and makes it possible, for the first time, to study several Greek traditional (autochthonous) wheat species and varieties and compare them with several commercial cereals, as well as other common beans and seeds. As previously reported, huge differences were observed among whole-grain and refined flours and respective final food products; interestingly, the grains of some local wheat varieties showed some of the highest reported concentrations.

## 2. Materials and Methods 

### 2.1. General

All solvents were of analytical grade (Merck). Syringaldehyde (98% purity, Sigma-Aldrich, Steinem, Germany) was used as internal standard (IS). IS solution was prepared in DMSO-d_6_ at a concentration of 0.5 mg/mL and kept at 4 °C. The IS solution was left to reach room temperature prior to use. The quantitative determination of alkylresorcinols was performed using NMR spectroscopy with a Bruker Avance DRX 400 MHz. The ^1^H-NMR spectra were processed using either the MNova (Mestrelab Research) or the TOPSPIN software (Bruker, Billerica, MA, USA). 

### 2.2. Plant Material and Processed Products

Several types of flour (white or whole-grain) originating from different wheat species, i.e., *Triticum dicoccum*, *T. monococcum*, *T. aestivum*, *T. durum* and *T. turgidum*, and other cereals, i.e., *Zea mays* (corn), *Hordeum vulgare* (barley), *Secale cereale* (rye), *Avena sativa* (oat) as well as the grains of the above plants and processed products (bread, flakes, wafers, pasta) and seeds of *Lens culinaris*, *Lathyrus clymenum*, *Phaseolus vulgaris*, *Cicer arietinum*, *Linum usitatissimum* and *Fagopyrum esculentum* were obtained from a certified producer in Greece (Antonopoulos farm, Dilofo). All plants were grown in the same location and the same year to reduce the impact of pedoclimatic factors.

### 2.3. Extraction and Isolation

*T. dicoccum* whole-grain flour (100 g) was extracted in an ultrasonic bath for 20 min with CH_2_Cl_2_ (300 mL). The solvent was evaporated and the residue was submitted to column chromatography using silica gel with cyclohexane and mixtures of cyclohexane with ethyl acetate (95:5, 90:10 and 85:15). Fractions obtained with cyclohexane-ethyl acetate 85:15 were pooled and submitted to preparative TLC. The alkylresorcinol spot was identified by the intense red color appearing after spraying with vanillin in methanol/sulfuric acid and heating. The spot with Rf = 0.32 (cyclohexane-ethyl acetate-acetic acid 80/20/1) was extracted with dichloromethane and analyzed by GCMS and ^1^H-NMR in CDCl_3_, DMSO, pyridine and acetone.

### 2.4. GC-MS

GCMS was performed using a Hewlett Packard 6890-5973 GC-MS system operating in EI mode (equipped with a HP 5MS 30 m × 0.25 mm, 0.25 μm film thickness capillary column). Helium (1 mL/min) was used as carrier gas. The initial temperature of the column was 60 °C; it was then heated to 280 °C at a rate of 3 °C/min.

### 2.5. High-Throughput Extraction and NMR Analysis

A portion of the flour or the comminuted corresponding product (330 ± 0.1 mg) was extracted in an ultrasonic bath for 5 min with deuterated DMSO (1 mL) containing syringaldehyde (0.5 mg/mL) as an internal standard. The supernatant liquid part was obtained by centrifugation (5 min at 3000 rpm) and was directly transferred to a 5 mm NMR tube. Each sample was analyzed in triplicate using a standard 90 degree excitation pulse, with a pulse width of 10 μsec and a prescan delay of 6.5 μsec. All measurements were performed at 298 K. Typically, 16 scans were collected into 32 K data points over a spectral width of 0–13 ppm (5263.18 Hz) with a relaxation delay of 10 s, an acquisition time of 3.11 sec and a FID resolution of 0.32 Hz. The appropriate relaxation delay was determined by gradual increases (1, 2, 5, 8, 10, 15, 20 s until the ratio between the integration of the peak of internal standard and the peak of the target compounds remained unchanged. The matching, tuning, shimming, receiver gain adjustment as well as phasing and baseline correction were always first performed automatically and then manually to achieve the best result. Prior to Fourier transformation (FT), an exponential weighting factor corresponding to a line broadening of 0.3 Hz was applied. For the peaks of interest, accurate integration was performed manually. The concentration of ARS was measured by comparing the area of the selected signal at 6.00 ppm with that of the internal standard (IS) at 9.79 ppm, which was set as 1. The calculation of the concentration of total ARS in mmol/mg of dry material was performed using the following formula: C = [(I_ARS_/3) × mmol _I.S._]/M_sample_, where M_sample_ = 330 mg and mmol I.S. = 0.5 mg/MW _syringaldehyde_ = 0.00270. To express the concentration in mg ARS/mg of dry material, we used the average molecular weight between C19 (*m*/*z* 376) and C21 (*m*/*z* 404) alkylresorcinols.

### 2.6. Precision-Recovery-LOD-LOQ 

The recovery was calculated by the comparison of successive extractions of samples for the studied ARS. The samples contained different levels of extractives in order to obtain unbiased results. The recovery achieved after one extraction was >95%. The intraday and interday precision was determined by analyzing three replicates of three random samples from the same day and from three different days, and the %RSD was found to be <10%. LOQ = 15 μg or 45 mg/Kg (S/N > 10) and LOD = 10 μg or 30 mg/Kg (S/N > 3).

## 3. Results

### 3.1. Comparison among Flour Extracts

**Figure 1:**
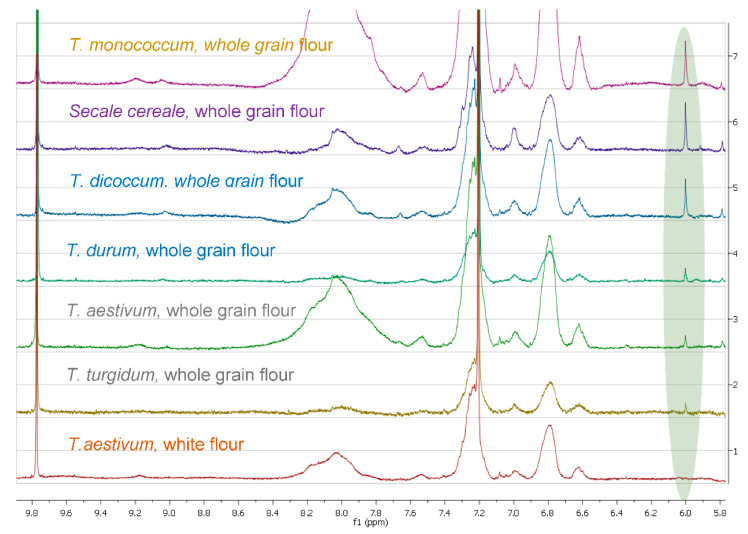
^1^H-NMR spectra in DMSO-d_6_ of flour extracts. The highlighted peak corresponds to the total 5-alkylresorcinols.

**Figure A1:**
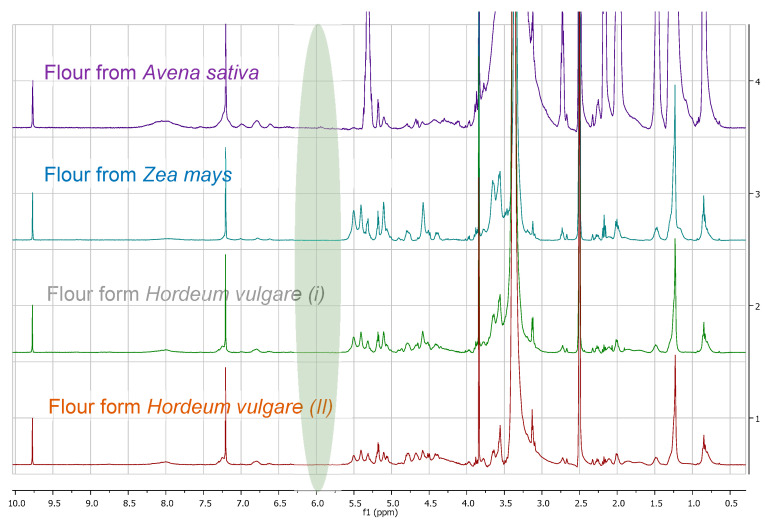
NMR spectra in DMSO from cereal flours without ARS.

**Figure A2:**
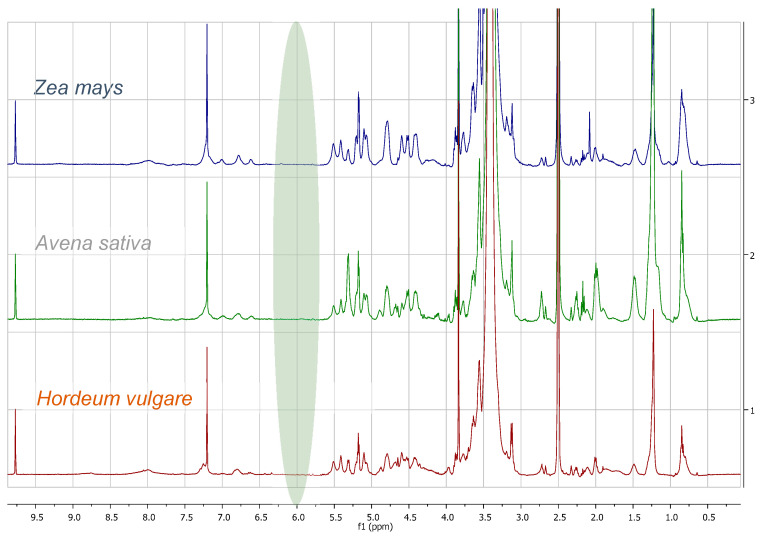
NMR spectra in DMSO from cereal grains without ARS.

**Figure A3:**
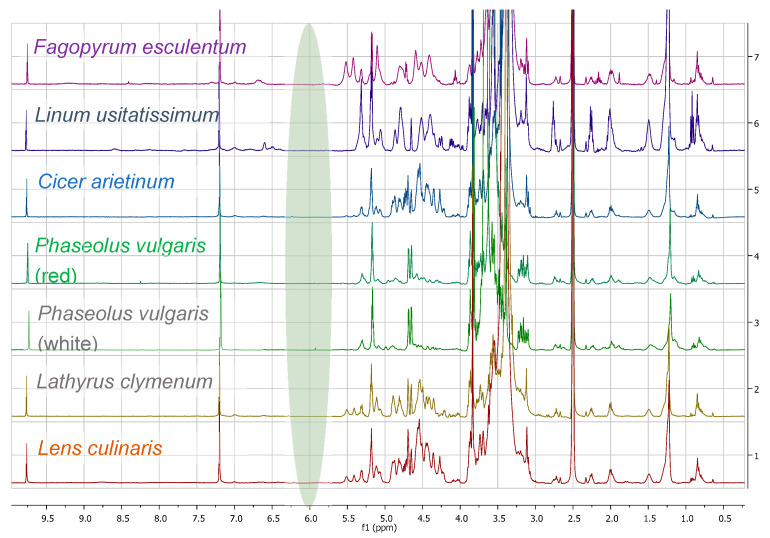
NMR spectra in DMSO from seeds and beans without ARS.

The obtained extracts of flours using DMSO-d_6_ were directly recorded by ^1^H-NMR without any other treatment. The spectra were compared and a singlet was observed at 6.0 ppm only in wheat and rye samples (Figure 1 and Figure A1, Figure A2, Figure A3). Interestingly, it was not observed in refined (white) flours.

**Figure A4:**
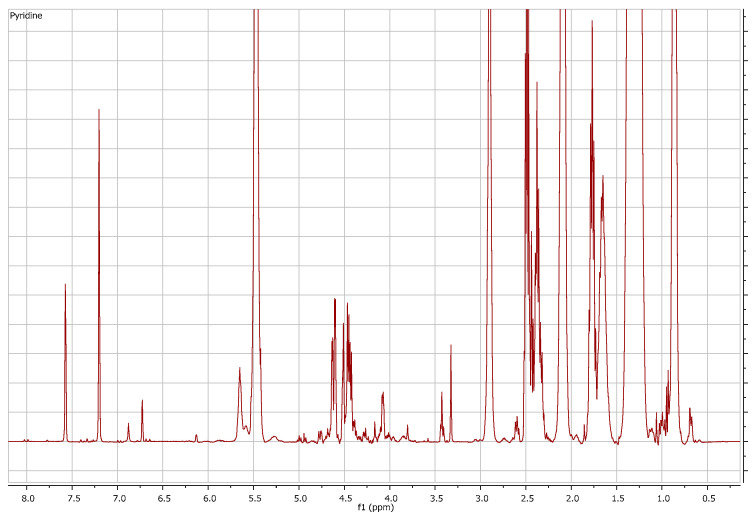
NMR spectrum of flour extract in Pyridine-d5.

**Figure A5:**
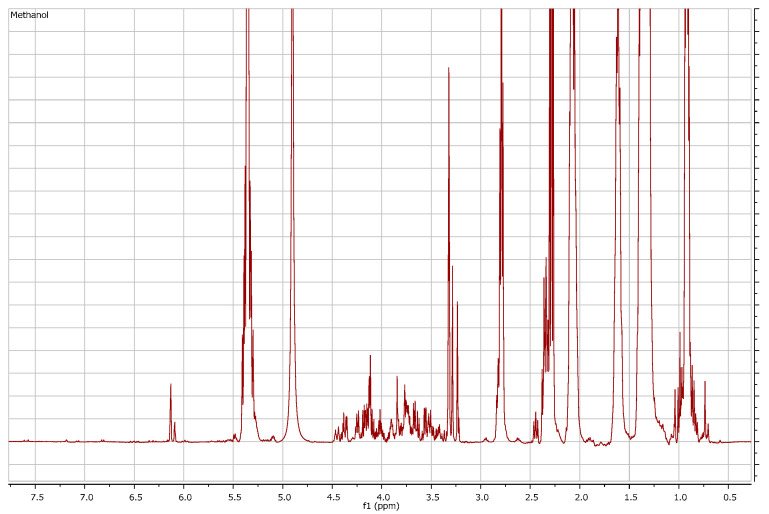
NMR spectrum of flour extract in CD_3_OD.

The same flour samples when extracted in other deuterated solvents showed more complicated spectra which precluded accurate quantitation (Figure A4 and Figure A5).

### 3.2. Structure Elucidation Results

**Figure 2:**
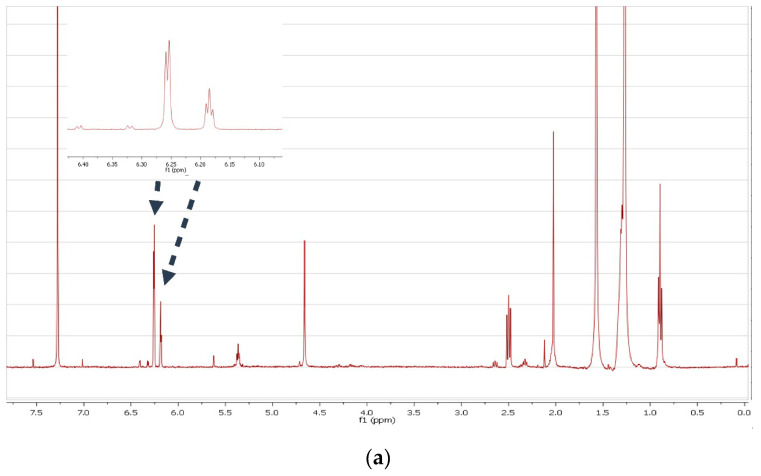

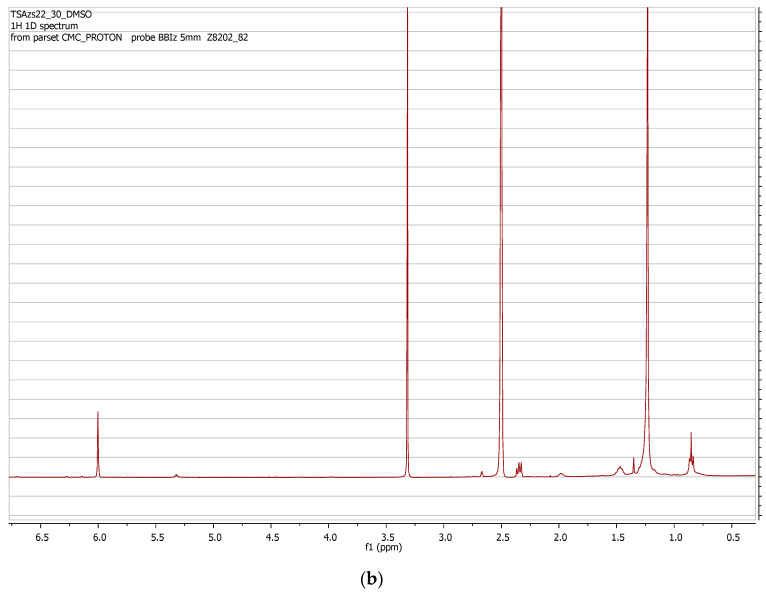
^1^H-NMR spectrum of the fraction containing the isolated ARS in CDCl_3_ (**a**) and in DMSO (**b**).

Targeted fractionation and isolation of the molecules related with the peak at 6.0 ppm showed that it corresponded to 5-alkylresorcinols. The NMR spectrum in CDCl_3_ (Figure 2) was similar with that described in literature [12]. Interestingly, the spectrum of the same compound in DMSO (Figure 2) showed only a single peak in the aromatic region, consistent with that observed in the flour extract.

**Figure 3:**
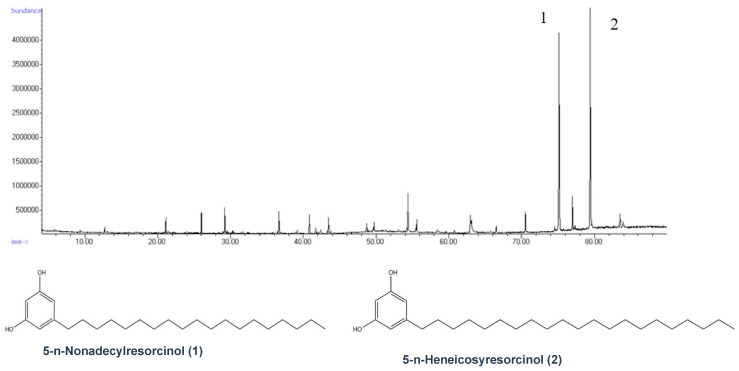
GC chromatogram of the isolated compounds.

A GCMS analysis of the isolated compound showed that it was a mixture of 5-n-alkylresorcinols with a side chain ranging from C15 to C25, but predominantly from C19 and C21 (Figure 3).

### 3.3. Quantitation Results

Grains, whole grain flours and breads, especially from old varieties of *Triticum monococcum* and *T. dicoccum*, showed high alkylresorcinols (ARS) contents (>450 mg/Kg), while the compounds were completely absent from white flour and all the commonly consumed corresponding types of bread.

We also found a high total alkylresorcinol content only in samples of *Secale cereale* flour, while no 5-alkylresorcinols could be detected in *Hordeum vulgare (barley)* flour, *Avena sativa (oat)* whole grain flour and flakes, *Zea mays* whole grain flour, nor in the seeds of *Lens culinaris, Lathyrus clymenum, Phaseolus vulgaris, Cicer arietinum, Linum usitatissimum, Fagopyrum esculentum* and *Zea mays*. The quantitation results are presented in Table 1.

## 4. Discussion

The main objectives of the current work were to investigate the possible differences among different types of flours from traditionally preserved Greek indigenous varieties of cereals, and especially those of wheat, and in parallel, to correlate those differences with the presence of bioactive natural ingredients. Based on the previous successful use of ^1^H-NMR for the qualitative and quantitative characterization of bioactive ingredients in olive oil, wine and beer [21,22,23], we first studied the total profile of selected flour extracts using microextraction with deuterated solvents and directly recorded the NMR spectra. 

After several trials of extraction and simultaneous spectra recording with CDCl_3_, CD_3_OD, Pyridine-d_5_, DMSO-d_6_ and Acetone-d_6_, we found that DMSO was the most efficient solvent, presenting in parallel the clearest spectra concerning the aromatic area which showed the most significant differences. A comparison among the different spectra (Figure 1) of the flours extracted with DMSO-d_6_ revealed the presence of a major peak in the aromatic area (6.0 ppm) only in rye and the wheat species. Targeted isolation of the molecules corresponding to this peak using liquid chromatography and subsequent characterization by NMR and GCMS led us to attribute it to a series of 5-alkylresorcinols. Interestingly, when the NMR spectrum of the isolated compound was recorded in CDCl_3_, Acetone-d_6_ or Pyridine-d_5_, it presented a doublet and triplet, as described in the literature for 5-alkylresorcinols [12], corresponding to the three aromatic protons (Figure 2A); however, when it was recorded in DMSO, the spectrum was simplified, presenting only one singlet in the aromatic region (H-2,4,6) (Figure 2B). In contrast to our results, the three protons H-2,4,6 of 5-alkylresorcinols in CDCl_3_ [27] were reported as two singlets, and in DMSO [28] as a doublet and a triplet. An analysis of the isolated compound by GCMS revealed that it was a mixture of several alkylresorcinols with side chains ranging from C15 to C25, but predominantly from C19 and C21 (Figure 3).

The simplicity of the NMR spectrum of the total extract in DMSO and the lack of overlapping peaks from other compounds allowed us to develop a high throughput quantitative analytical method for the measurement of total alkylresorcinols. The method was based on the integration of the peak at 6.0 ppm (I_ARS_) and comparisons with the integration of the internal standard at 9.79 ppm, which was set as 1. The calculation of the concentration of total ARS in mmol/mg of dry material was performed using the formula presented in Materials and Methods, which was based on the molar ratio between the target analyte and the internal standard, following the general guidelines presented by Pauli et al. [29] and Bharti and Roy [30]. The integration value of the singlet at 6.0 ppm was divided by 3, since it corresponded to three aromatic protons.

**Figure A6:**
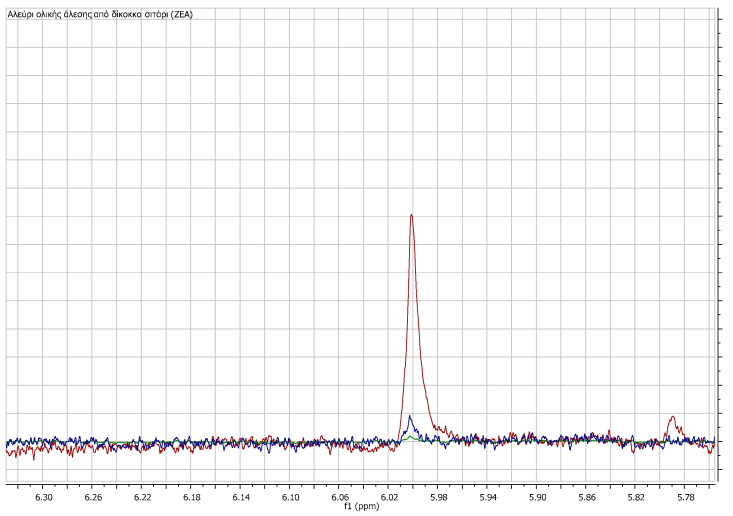
^1^H NMR spectrum of whole grain flour from Triticum dicoccum (ZEA^®^), after three successive extractions (red line 1st extraction, blue line 2nd extraction, green line 3rd extraction).

It should be noted that one extraction step in an ultrasonic bath for 5 min is sufficient for the quantitative recovery (>95%) of ARS. Longer extraction times or more than one cycle of extraction led to a negligible increase of the recovery, i.e., less than 5% (Figure A6).

*Advantages of the qNMR method in comparison to chromatographic methods*

Alkylresorcinols (ARS) have been previously investigated and quantified in several wheat products, mainly using time-consuming chromatographic methods and long extraction or pretreatment procedures [4,5,6,7,8,9,10,11,12,13,15,16,17,18,19,20]. Depending on the analyzed material (flour, grain, bread or pasta) and the analytical method (liquid or gas chromatography or colorimetry), several methods of extraction have been used, most commonly with ethyl acetate, acetone, hot propanol or propanol/water, with extraction times ranging from 3 to 48 h, followed by several filtration, evaporation and dilution or derivatization steps. In all cases, external standards of one or more ARS are necessary. Existing chromatographic methods (GCMS, HPLC-UV/DAD or LCMS) give very good results but the time of analysis ranges from 15 min to 90 min. Colorimetric methods require times ranging from 15 min to 4 h.

Our new method is the first to make possible the selective and high throughput quantitative determination of ARS in grains, flours and foods in less than 15 min per sample, including the extraction and analysis time. In addition, the most important advantages are: (i) no need for standards for target compounds; (ii) no need for derivatization; (iii) short time of extraction in ultrasonic bath (5 min); (iv) very simple procedure for the separation of the solution for analysis by centrifugation; and (v) adequate limit of detection and precision. The only limitation is that the current method cannot determine the length of the side chain, which, in some cases, is useful to differentiate rye from wheat [4,5].

*Comparison among the studied cereals and other common seeds and beans*

As previously reported, ARS were found only in wheat and rye, and not in other cereals. Indeed, analyses of the grains of the studied cereals, as well as several of other commonly used beans and seeds, confirmed that the main nutritional source of ARS is wheat and rye. Wheat should be considered as a primary nutritional source, especially for Mediterranean populations, since rye is consumed to a lesser extent. Until the mid-20th century, the main flour used for bread preparation was whole-grain wheat flour, usually coming from local varieties, and consequently, the traditional Mediterranean diet should be considered as a diet that is very rich in ARS. In modern days, the massive use of white (refined) flour from *T. aestivum* has significantly reduced the nutritional intake of ARS in Mediterranean populations. 

*Comparison among wheat species and previously reported ARS content in wheat grains and flour*

**Figure A7:**
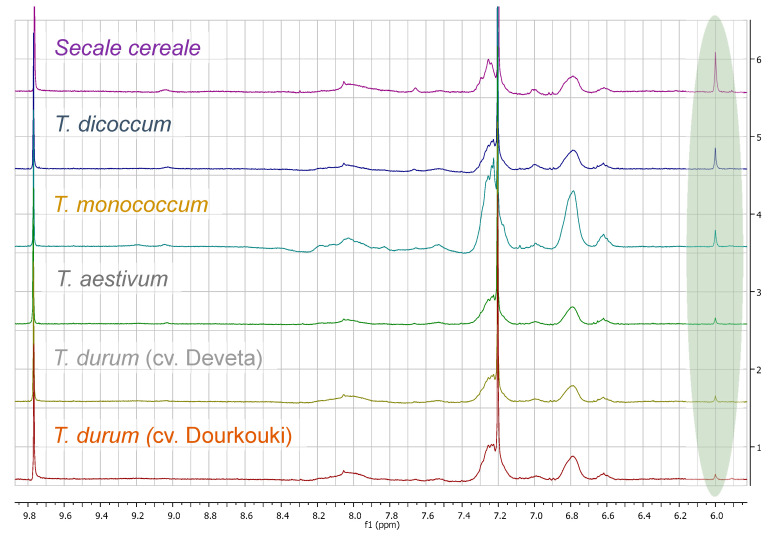
^1^H-NMR spectra in d_6_-DMSO of extracts of grains showing the ARS peak.

In our study, the local, traditional variety of *T. monococcum* (known as “Kaploutzas”) showed the highest content of ARS in whole grain flour, i.e., even higher than the corresponding rye flour. It showed a small difference in comparison with *T. dicoccum* and a big difference with *T. durum, T. aestivum* and *T. turgidum*. Interestingly, the analysis of the grains showed that *T. dicoccum* presented the highest content of ARS (>1000 mg/Kg), close with that of *T. monococcum*, but again, very different from those of *T. aestivum* and *T. durum.* The results obtained herein are in agreement with previous analyses [4,6,15] concerning whole grain and refined white flours. Previous studies have also shown that the grains of *T. monococcum and T. dicoccum* are richer than *T. aestivum* and *T. durum*, but herein, the concentrations in local varieties of *T. monococcum* (925 mg/Kg) and *T. dicoccum* (1148 mg/Kg) were found to be much higher than what was previously reported from Germany and Hungary (391–819 mg/Kg) [4,8,17] and also from all other wheat grains reported until now, with the exception of *T. timophevi* (Figure A7). A comparative list of all previous studies measuring the levels of ARS in wheat grains and products is presented in Table A1. 

*ARS content of bread, pasta, wafers*

**Figure A8:**
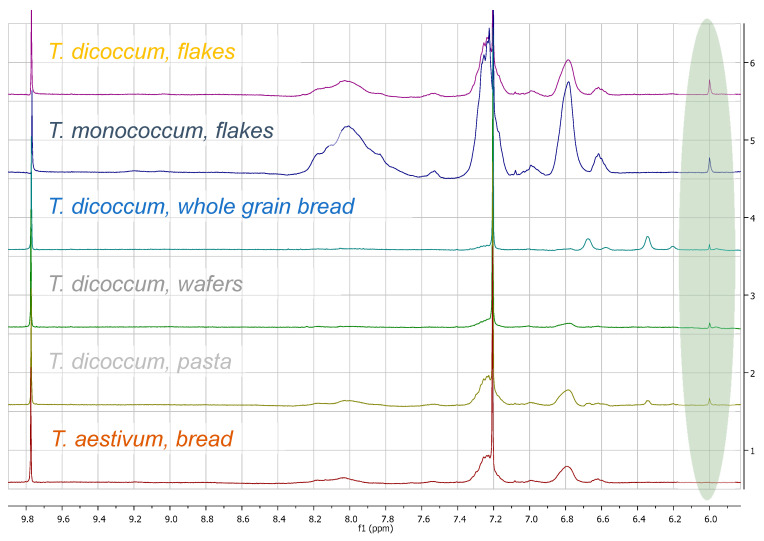
^1^H-NMR spectra in d_6_-DMSO of extracts of wheat products showing the ARS peak.

The results obtained for *T. dicoccum* grains and the fact that this species is becoming popular again for baking prompted us to study the fate of ARS during the production of bread, flakes, wafers and pasta. Interestingly, all products contained significant amounts of ARS (Table 1, Figure A8). The flakes which has undergone the least processing, as expected, showed the highest content. Bread retained the majority of ARS during baking, in contrast to previous studies showing that ARS disappeared during baking. In fact, DMSO extraction and the applied analysis methods probably aided in overcoming the problems of previous methods, where interactions with starch led to poor results.

## 5. Conclusions

Alkylresorcinols are a class of major bioactive ingredients present in wheat flour and wheat-based products, and found in high amounts in old wheat varieties while being almost absent from the common white flours that are used for the mass production of bread nowadays. Their concentration can easily be measured by qNMR, permitting high-throughput analyses of flour and related products. 

## Figures and Tables

**Table 1:** Concentration of total alkylresorcinols in flours, grains and processed products.

Sample	ARS mg/Kg	ARS mmol/Kg
**Flours**		
*T. monococcum,* whole-grain flour	638 ± 32	1.64 ± 0.08
*Secale cereale,* whole-grain flour	574 ± 23	1.47 ± 0.06
*T. dicoccum,* whole-grain flour	510 ± 30	1.31 ± 0.07
*T. durum,* whole-grain flour	191 ± 6	0.49 ± 0.02
*T. aestivum,* whole-grain flour	121 ± 5	0.33 ± 0.01
*T. turgidum,* whole-grain flour	63 ± 3	0.16 ± 0.01
*T. aestivum,* white-flour	ND	ND
**Wheat grains**		
*T. dicoccum*	1148 ± 58	2.95 ± 0.15
*T. monococcum*	925 ± 74	2.37 ± 0.19
*T. aestivum*	255 ± 23	0.65 ± 0.06
*T. durum* (cv.deveta)	255 ± 12	0.65 ± 0.03
*T. durum* (cv.dourouki)	191 ± 4	0.49 ± 0.01
**Processed products**		
*T. dicoccum* Bread from whole-grain flour	455 ± 32	1.17 ± 0.08
*T. dicoccum* Flakes	1090 ± 54	2.79 ± 0.14
*T. dicoccum* Wafers	351 ± 14	0.9 ± 0.04
*T. dicoccum* Pasta	330 ± 10	0.85 ± 0.03
*T. monococcum* Flakes	1084 ± 97	2.78 ± 0.25
*T. aestivum* Bread from white flour	ND	ND

ND: not detected.

## Appendix A

Table A1 contains a comparative list of all previous studies measuring the levels of ARS in wheat grains and products. NMR spectra of analyzed seeds and beans are provided as Figure A1, Figure A2 and Figure A3. Examples of NMR spectra of extracts recorded in solvents other than DMSO are provided as Figure A4 and Figure A5. NMR spectra showing the recovery of each extraction step are provided as Figure A6. NMR spectra of wheat grains and products are provided as Figure A7 and Figure A8.

**Table A1:** Alkyresorcinols content in grains, flours and related products from wheat species reported in literature between 1988 and 2020.

Product	*Triticum* spp.	Origin	ARs Content (mg/kg)	Reference
whole-wheat flour	*Triticum* spp.	Finland	759	[6]
white wheat flour			47	
white wheat flour, organic			44	
wheat bran			3225	
white wheat bread			nd	
pasta			48	
	*T. durum*	France, Germany, and United Kingdom	687	[4]
	*T. aestivum*		909	
	*T. spelta*		819	
	*T. timophevi*		1480	
	*T. compactum*		1090	
	*T. ispahanicum*		982	
	*T. polonicum*		951	
	*T. paleocolchicum*		916	
	*T. carthlicum*		876	
	*T. sphaerococcum*		862	
	*T. dicoccum*		819	
	*T. dicoccoides*		787	
	*T. orientale*		691	
	*T. araraticum*		599	
	*T. turanicum*		200	
Commercial wheat bran	*Triticum* spp.	Uppsala, Sweden	2672	
Commercial wheat bran based cereal			1784	
Commercial wheat breakfast biscuits			558	
Commercial whole grain wheat flour			550	
Commercial crusty whole grain wheat bread			222	
Commercial wholemeal wheat crackers			179	
Commercial wholemeal wheat bread			142	
Commercial wheat crispbread			58	
Commercial digestive biscuits			57	
Commercial white wheat flour			nd	
Commercial white wheat bread			nd	
refined wheat	*Triticum* spp.	US	20.9	
semolina	*T. durum*	US	71.3	[17]
whole grain	*T. durum*	US	444.2	
whole grain	*T. monococcum*	Germany	391	
whole grain	*T. turanicum*	Germany	255.5	
refined wheat flakes + bran	*Triticum* spp.	France	100.7	
white wheat bread		US	19.8	
white wheat bread		France	28	
refined wheat tortilla		US	13.6	
refined wheat crackers		US	16.8	
refined wheat dry toast		Italy	34.2	
WG wheat bread		US	277.5	
WG wheat roll		US	467	
WG wheat dry toast		Italy	193	
WG wheat dry toast		France	192.8	
refined wheat pasta		US	44	
refined wheat pasta (raw)		US	53.5	
refined wheat pasta (cooked)		US	58	
refined wheat pasta		Italy	49.5	
WG wheat pasta		Italy	201.2	
WG wheat pasta		US	237.5	
WG wheat pasta 4 (raw)		US	348.9	
WG wheat pasta 4 (cooked)		US	380.3	
WG wheat pasta		Netherlands	383.1	
WG wheat pasta		Italy	214.2	
Kernel	*T. durum*	Austria	251–475	[10]
		France	447–467	
		Kazakstan	488–618	
		Russia	483	
		Spain	401–526	
		Sweden	407–490	
Commercial pasta		Sweden	215–270	
Kernel	*T. aestivum spring*	Germany	663–862	[18]
	*T. aestivum winter*		591–1077	
	*T. durum spring*	Austria	430–797	
	*T. durum winter*		554–675	
Whole Grain	*T. aestivum winter*	Sweden	432–634	[5]
	*T. aestivum spring*		227–353	
wheat bran	*T. aestivum*		2211 ± 605	
white wheat flour	*T. aestivum*		nd	
Commercial white bread	*Triticum* spp.	Latvia	24.7–26.8	[19]
Commercial white bread with seeds			26.4–27.7	
Commercial white bread		Finland	24.9–30.8	
whole-grain wheat	*T. aestivum*	Poland	586–943	[7]
whole-grain wheat ground			565–879	
wheat bran			2388–3186	
Commercial whole-wheat flour	*Triticum* spp.		258–269	
Commercial breakfast wheat cereals			131–671	
Commercial soft wheat bread			0–23	
bread	*T. spelta*		390	
whole-grain bread	*Triticum* spp.		140–497	
Wheat Grain	*T. spelta*	Hungary	427–605	[8]
	*T. durum*		327–399	
	*T.monococcum*		399–595	
	*T.dicoccum*		444–581	
	*T. aestivum* spring		341–416	
	*T. aestivum* winter		340–410	
Grains	*T. durum*	Hungary	370 ± 100–452 ± 138	[16]
	*T. aestivum* spring		494 ± 79–536 ± 111	
	*T. aestivum* winter		500 ± 114–655 ± 142	
Grains	*T. aestivum winter*	Sweden	600	[1]
Bran	*T. aestivum*	China	697–1732	[13]
White flour	*T. spelta*	China	31.8	[15]
organic white wheat flour	*Triticum* spp.	China	26.3	
white wheat flour		China	29.9–34.7	
white wheat flour		Sweden	13.5–27.6	
semolina		China	46.6	
brown wheat flour		China	98.1- 215.6	
Whole grain wheat flour		China	285.4–660.3	
organic whole grain wheat flour		China	488.9	
Whole grain wheat flour		India	138.4–345.5	
Whole grain white wheat		China	555.5	
grains	*T. spelta*	China	455.3	
flour	*T. spelta*	Sweden	507.3	
Whole grain wheat pasta		China	40.8	
Whole grain wheat pasta	*Triticum* spp.	Italy	313.7–340.5	
Whole grain wheat pasta		United Kingdom	422.8–608.5	

nd: non detected.
